# Hierarchical Microstructure–Mechanical Property Correlations in Superior Strength 5 wt% Cr Cold-Work Tool Steel Manufactured by Direct Energy Deposition

**DOI:** 10.3390/ma18133113

**Published:** 2025-07-01

**Authors:** Jung-Hyun Park, Young-Kyun Kim, Jin-Young Kim, Hyo-Yun Jung, Sung-Jin Park, Kee-Ahn Lee

**Affiliations:** 1 Department of Materials Science and Engineering, Inha University, Incheon 22212, Republic of Korea; pjhhemuri@inha.edu (J.-H.P.); ykkim@kims.re.kr (Y.-K.K.); 2 Advanced Metals Division, Korea Institute of Materials Science (KIMS), Changwon 51508, Republic of Korea; 3 Engineering Center, Hanwha Aerospace, Changwon 51542, Republic of Korea; zzin81@gmail.com; 4 Advanced Process and Materials R&D Group, Korea Institute of Industrial Technology, Incheon 21999, Republic of Korea; hjung@kitech.re.kr; 5 Shin Young Corp., Yeongcheon 38899, Republic of Korea; sjpark92@shym.co.kr

**Keywords:** direct energy deposition, 5 wt% Cr cold work tool steel, microstructure, mechanical property, post-heat treatment, strengthening mechanism

## Abstract

The direct energy deposition (DED) metal additive manufacturing process enables rapid deposition and repair, providing an efficient approach to producing durable tool steel components. Here, 5 wt% Cr cold-work tool steel (Caldie) was developed by reducing carbon and chromium to suppress coarse carbide formation and by increasing molybdenum and vanadium to enhance dimensional stability. In this study, Caldie tool steel was fabricated via DED for the first time, and the effects of post-heat treatment on its hierarchical microstructure and mechanical properties were investigated and compared with those of wrought (reference) material. The as-built sample exhibited a mixed microstructure comprising lath martensite, retained austenite, polygonal ferrite, and carbide networks, which transformed into full martensite with fine carbides after heat treatment (DED-HT). The tensile strength of the DED Caldie material increased from 1340 MPa to 1949 MPa after heat treatment, demonstrating superior strength compared to other heat-treated, DED-processed high-carbon tool steels. Compared to DED-HT, the wrought material exhibited finer martensite, a more uniform Bain group distribution, and finer carbides, resulting in higher strength. This study provides insights into the effects of heat treatment on the hierarchical microstructure and mechanical behavior of Caldie tool steel manufactured by DED.

## 1. Introduction

Cold-work tool steels offer excellent hardness, toughness, strength, and wear resistance, rendering them suitable for a wide range of industrial applications. Among these, 5 wt% Cr cold-work tool steel (Uddeholm Caldie) is known for its exceptional resistance to chipping and cracking as well as its high strength, making it ideal for use in cold-work parts, such as blanking dies, machine knives, rolling dies, and cold forging and trimming operations [1,2,3]. Compared with other high-carbon, high-chromium tool steels, Caldie materials have reduced carbon (0.7–0.8 wt%) and chromium (4.5–5.0 wt%) contents to mitigate the adverse effects of coarse carbides. Conversely, the molybdenum and vanadium contents are increased to precipitate various small carbides during heat treatment, forming uniform and fine-tempered martensite to improve the dimensional stability [4,5].

Metal additive manufacturing process technology holds great potential for a wide range of industries because it enables the immediate manufacture of optimized part geometries. Among the various metal additive manufacturing (AM) methods, direct energy deposition (DED), which is based on laser cladding engineering, uses an energy source to precisely deposit the feedstock onto a substrate. This method has the advantage of rapid manufacturing of parts with fewer size limitations [6,7,8,9]. Therefore, DED has shown promise in tool and die industries, where these advantages are most needed. Compared to other AM techniques, such as laser powder bed fusion (LPBF) or electron beam melting (EBM), the DED process offers faster deposition rates, stronger metallurgical bonding to the substrate, and flexibility for localized repair or hardfacing applications, which are advantageous for extending the service life of tool steel components [10,11]. Additionally, due to relatively high cooling rates than conventional manufacturing methods, DED promotes a high degree of solid solution of alloying elements in the martensite matrix, enhancing hardness and wear resistance [12]. However, the DED process is also subject to repeated thermal cycling, which may cause liquid cracking or microstructural heterogeneities, particularly in high-carbon tool steels [13]. These aspects must be carefully managed to ensure consistent part quality and mechanical performance.

The DED process can produce tool steel materials with microstructural properties that differ from those produced via conventional processes. These microstructural differences alter the mechanical properties [14,15]. Owing to the high cooling rates generated using the DED process, thermal gradients and in situ tempering effects can result in cellular or columnar dendrite structures [16]. Furthermore, microsegregation of alloying elements can lead to the formation of multiphase structures and small precipitates [17]. Recent studies have also emphasized the importance of microstructural factors in improving the mechanical properties of tool steel alloys produced by the DED process [17,18].

The application of post-heat treatments comprising austenitizing and tempering along with microstructural control is necessary to impart good mechanical properties to high-carbon tool steels. Controlling the post-heat treatment conditions mitigates chemical inhomogeneities and creates a fine grain, resulting in an optimized combination of mechanical properties. The hierarchical microstructure, characterized by martensite packets, blocks, and laths within the prior austenite grain, along with precipitation strengthening mechanisms, has a strong influence on mechanical properties, such as impact toughness and dynamic tensile fracture [19,20,21,22]. In other words, controlling the size, shape, and orientation of the hierarchical microstructure formed using post-heat treatment in combination with carbides or intermetallics can improve the resistance to loading by hindering dislocation migration or increasing the uniform deformation by effectively absorbing and redistributing energy under a load. Recent studies have reported the effects of post-heat treatment on high-carbon tool steel materials manufactured using the DED process. Shim et al. showed that for AISI M4 (Fe-4Cr-4Mo-4V-5W) tool steel manufactured by DED, the impact toughness increased and the wear resistance decreased in the post-heat-treated material compared to the as-built material [23]. These results were attributed to the reduction in residual stress and the formation of small carbides owing to the application of post-heat treatment. Chen et al. applied a post-tempering annealing treatment to crucible particle metallurgy material manufactured by the DED process and found the best hardness value at the 500~550 °C condition [24]. Moreover, the effect of small carbide formation and growth was greater than that of retained austenite changes with changes in the tempering temperature. Jeong et al. observed that a phase change from austenite to martensite occurred, and the morphology of the carbide changed from the spherical/rod type to the agglomerated eutectic type upon the application of the post-heat treatment to Fe-8Cr-3V-2Mo-2W tool steel prepared by DED [25]. Grain refinement due to martensitic transformation increased the strength and wear resistance, whereas agglomerated carbides that precipitated from the grain boundaries reduced the toughness through stress concentration. Park et al. applied a post-heat treatment to AISI D2 materials produced by DED and forging processes and reported that DED materials consisting of uniformly dispersed fine carbides had relatively low anisotropic characteristics, whereas wrought materials consisting of coarse carbides oriented in the forging direction [26]. However, there have been significantly fewer studies on high-carbon tool steels manufactured using DED than on low- and medium-carbon tool steels. This is due to the fact that the high carbon content in the tool steel increases crack susceptibility, which poses a challenge for additive manufacturing [27].

Caldie steel offers excellent mechanical properties, availability, accessibility, and competitive pricing. This could provide attractive guidelines for its application in the high-carbon tool steel sector, if successfully manufactured using the DED process. In addition, the microstructure and properties of tool steel parts manufactured by DED can be presented more objectively than those of conventional materials under the same conditions. However, to date, no studies have reported on the properties of 5 wt% Cr cold-work tool steel manufactured using the DED process.

In this study, crack-free 5 wt% Cr cold-work tool steel (Uddeholm Caldie, Hagfors, Sweden) was fabricated for the first time using a DED additive manufacturing process. The effect of the post-heat treatment on the mechanical properties of the fabricated material was investigated. The as-built microstructural evolution process and tensile properties after post-heat treatment were examined, and the influence of the hierarchical microstructure on the deformation behavior and fracture mechanisms was analyzed. Moreover, the mechanical properties and microstructural characteristics were compared with wrought materials subjected to the same post-heat treatment.

## 2. Materials and Methods

### 2.1. Materials and DED Processing Conditions

**Figure 1:**
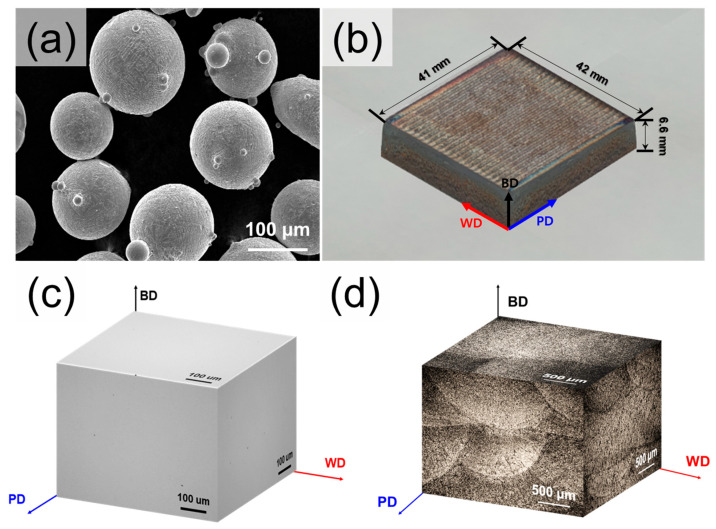
(**a**) SEM images of powders used in the current study; (**b**) macroscopic images of as-built samples manufactured by direct energy deposition. BD: building direction, PD: printing direction (parallel to the scanning direction), WD: width direction (perpendicular to the scanning direction), (**c**) Three-dimensional OM micrograph in as-built sample with high density; (**d**) three-dimensional OM micrograph in as-built sample after etching.

The powder morphologies used in this study are shown in Figure 1a. The powder size distribution was measured using a particle size analyzer (PSA, Mastersizer 3000, Malvern Panalytical, Worcestershire, UK), which resulted in particles in the size range of 36.3–154.8 μm, with an average particle size of 84.9 μm. To prepare the 5 wt% Cr cold-work tool steel in bulk form, DED was performed using an MDG-300 (Maxrotec, Daegu, Republic of Korea). The DED process was carried out using a laser (λ = 1070 nm) with a spot diameter of 1.5 mm, and the process was conducted in an argon atmosphere. The optimal process conditions for suppressing the pore formation were set to a laser power of 1400 W, a laser traverse speed of 500 mm/min, and a powder feed rate of 4 g/min. Each layer was deposited with a thickness of 500 µm. To reduce residual stress and enhance microstructural uniformity, a scanning strategy with 78° inter-layer rotation was implemented. To prevent cracking during the manufacturing process, a preheating system was applied to control the temperature by connecting the heating cartridge to the substrate (STS3 steel). A preheating temperature of 400 °C was applied to the substrate. This temperature was selected to exceed the martensite start temperature (Ms) of Caldie steel [5]. An initial attempt at 300 °C preheating resulted in the presence of internal cracks, whereas samples fabricated at 400 °C were free from observable cracking, confirming the effectiveness of this temperature setting.

Consequently, a bulk-shaped sample with dimensions of 41.0 mm × 42.0 mm × 6.6 mm was successfully fabricated (Figure 1b). The post-heat treatment process applied to the as-built (designation: DED-built) samples was based on the standard heat treatment (consisting of austenitization and tempering) provided by Uddeholm. Austenitization was performed at 1020 °C for 30 min followed by oil quenching. Double tempering was then performed at 520 °C for 2 h, followed by air cooling (designation: DED-HT). Both as-built and heat-treated samples were extracted from the same DED-fabricated bulk material. Heat treatment was carried out in a box furnace under an argon gas atmosphere to minimize oxidation during thermal exposure. For comparison, the same post-heat treatment was applied to 5 wt% Cr cold-work tool steel produced via the forging process (designation: Wrought-HT). The chemical compositions of DED and wrought materials were analyzed using an inductively coupled plasma–optical emission spectrometer (ICP-OES, Agilent 720, Agilent Technologies, Santa Clara, CA, USA) and a C/S analyzer (CS 884, Leco, St. Joseph, MI, USA); the results are presented in Table 1.

### 2.2. Microstructural and Solidification Behavior Analysis

For initial microstructure observation of the materials, the samples were polished using #400~#4000 SiC paper, 1 μm of diamond suspension, and 0.04 μm of colloidal silica solution. The polished samples were etched using Vilella’s reagent (1 g of picric acid, 4 mL of HCl, and 95 mL of ethyl alcohol) for 30 s. The matrix and precipitates were observed by scanning electron microscopy (SEM, S-4300, Hitachi, Tokyo, Japan). For carbide size measurement, ten SEM micrographs per sample condition were analyzed, with over fifty precipitates measured using image analysis software (Image-Pro Plus 7, Media Cybernetics, Rockville, MD, USA). The distribution of alloy elements in the specimens was measured using an electron probe micro-analyzer (EPMA, JXA-8530F Plus, JEOL, Tokyo, Japan). The phase and misorientation distributions of the initial and deformed samples were analyzed by electron backscatter diffraction (EBSD, Nordlys nano-detector, Oxford, UK) at an accelerating voltage of 20 kV, and OIM software (TSL OIM analysis 8, EDAX, Mahwah, NJ, USA) was used for data analysis. The EBSD maps were taken from the central region of a melt pool and from the mid-height and mid-thickness of the as-built sample to avoid local variations at melt pool boundaries and minimize the effects of thermal accumulation. High-resolution transmission electron microscopy (HRTEM, JEM-2200FS, JEOL, Tokyo, Japan) with a 200 kV field emission gun was used to analyze the small precipitates observed in the bright field and high-angle annular dark-field scanning transmission electron microscopy (HAADF-STEM) modes. The Scheil–Gulliver rapid solidification model was simulated using Thermo-Calc software (2019a).

### 2.3. Mechanical Properties

The Vickers hardness of the specimens was measured 12 times using a micro-Vickers hardness tester (HM-200, Mitutoyo, Kanagawa, Japan) with a 0.3 kgf load and 10 s holding condition, and the average value was used. The specimens for tensile testing were machined as plate dog-bone type tensile specimens with a gauge length of 12 mm, gauge width of 3 mm, and gauge thickness of 1 mm. Tensile specimens were extracted using electrical discharge machining (EDM). Owing to the limited build height of the as-built sample, the specimens were machined in a horizontal orientation (perpendicular to the build direction).

Tensile tests were performed in triplicate using an Instron 8501 tester (Norwood, MA, USA) at 1 × 10^−3^/s initial strain rate, and the average value was used. The elongation was calculated using the plastic strain at fracture method.

## 3. Results

### 3.1. Microstructures of DED and Wrought 5 wt% Cr Cold-Work Tool Steels

The areal density values of the DED-built sample, according to orientation, were 99.96 ± 0.02% (BD), 99.97 ± 0.02% (PD), and 99.95 ± 0.03% (WD), indicating high densities in all regions (Figure 1c). The areal density was evaluated from cross-sectional OM images of the sample, excluding the outermost 1 mm from each edge to minimize boundary effects. A total of 16 images were acquired at 200× magnification, and the average density was calculated based on the fraction of pores in the analyzed area. As shown in Figure 1d, the etched OM image of the DED-built sample exhibits distinct melt pool boundaries and fine dendritic substructures, which are typical microstructural features resulting from rapid solidification and repeated thermal cycling during DED.

**Figure 2:**
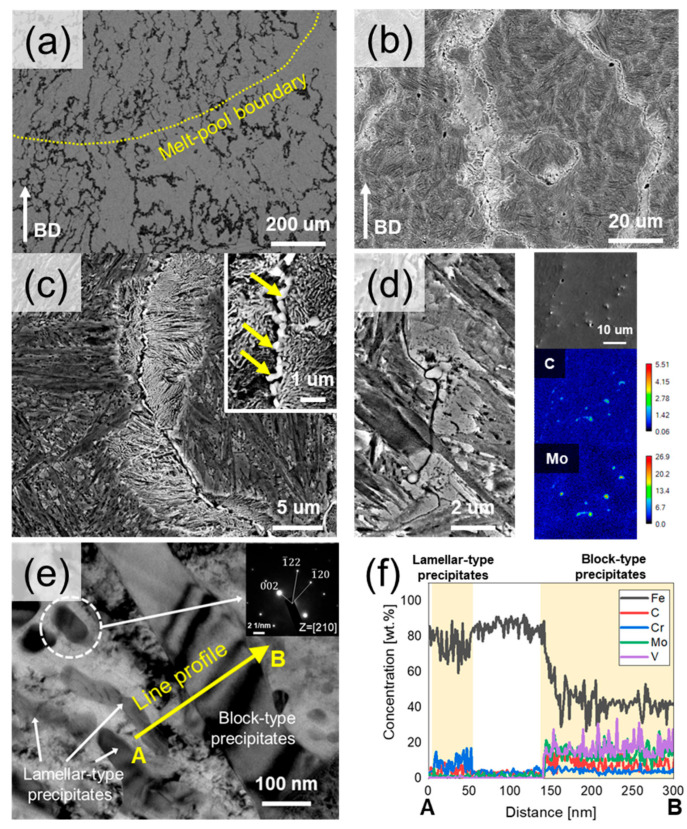
(**a**) Low magnification OM image of layer-wise microstructure. (**b**) SEM image showing initial microstructures of the DED-built sample after etching. (**c**) Precipitate network formed along the build direction, (**d**) Prior austenite grain boundary region with C and Mo microsegregation, (**e**) high-magnification image of lamellar and block precipitates, and (**f**) TEM-EDS line scanning results across the lamellar and block precipitates in (**e**).

Figure 2 shows the initial microstructure of the DED-built specimen after etching. The DED-built material exhibits a fine martensite structure within the prior grain boundaries and continuous network precipitates (Figure 2a,b). The network precipitates exhibit a unique structure, with lamellar-type precipitates distributed around the block-type precipitates (Figure 2c). Block-type precipitates (yellow arrow in Figure 2c) are 0.4~1.5 μm in size, whereas lamellar-type precipitate particles are 0.07~0.1 μm wide. It can be inferred that these two types of precipitates are microscopic because of the high cooling rate during the solidification process. In the prior grain boundary region, blocky carbides with a size distribution of 0.5~1.2 μm along the boundary are identified (Figure 2d). The microsegregation of carbon and molybdenum was observed along prior grain boundaries, as shown in the EPMA mapping (Figure 2d). Local carbon enrichment in these regions lowers the martensite start (Ms) temperature, thereby stabilizing retained austenite. The elemental distributions of the block- and lamellar-type precipitates in Figure 2e were confirmed using TEM-EDS analysis, and the results are shown in Figure 2f. The results show that C, Cr, Mo, and V are highly concentrated in the block-type precipitates, whereas C and Cr are highly concentrated in the lamellar-type precipitates, indicating that both precipitates are carbides. Furthermore, TEM analysis confirms the lamellar-type carbide to be Cr_7_C_3_.

**Figure 3:**
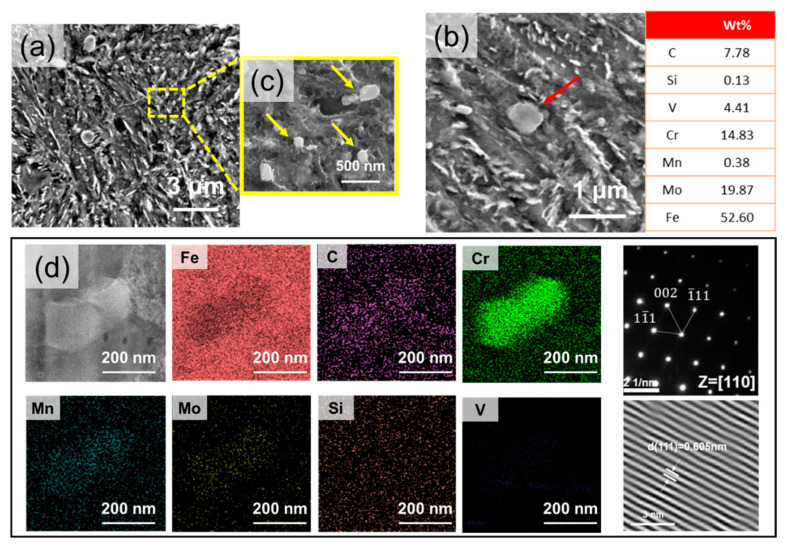
(**a**) SEM images showing initial microstructures of the DED-HT sample after etching, (**b**) type 1 precipitate (red arrow) and chemical composition of type 1 showing the Cr-, Mo-, and V-rich carbide, (**c**) type 2 precipitate (yellow arrow), and (**d**) STEM-EDS mapping results of the type 2 precipitates and HRTEM image with SAED pattern, indexed as Cr_23_C_6_.

In the DED-HT specimen, fine precipitates uniformly distributed within the matrix are observed and identified as two types, namely type 1 (Figure 3b) with a size of 0.70~1.20 μm and type 2 (Figure 3c) with a size distribution of 0.15~0.25 μm. The SEM images clearly show that the type 2 precipitates have a higher density distribution than that of the type 1 precipitates. TEM-EDS analysis reveals that the type 1 precipitates exhibit high C, Cr, Mo, and V contents, which are inferred to be the residual decomposition of the block-type carbide in the DED-built sample after post-heat treatment. Type 2 precipitates are identified as Cr-rich M_23_C_6_ carbides using TEM (Figure 3d). During heat treatment at high temperatures, the concentration gradient of C and Cr between the carbide and matrix can promote the diffusion of atoms from Cr_7_C_3_ into the matrix. Accordingly, the concentrations of C and Cr in the matrix increase, and the formation of Cr_23_C_6_ may increase after carbide nucleation during tempering [28].

**Figure 4:**
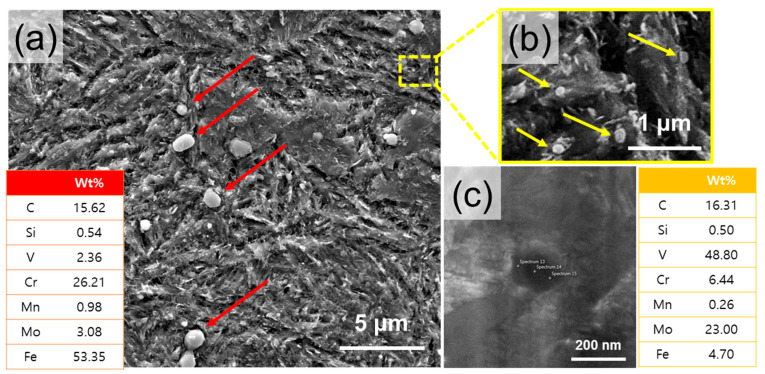
(**a**) SEM images showing initial microstructures of the Wrought-HT sample after etching with type 3 precipitate (red arrow) and the chemical composition of type 3 showing the Cr-rich carbide. (**b**) Type 4 precipitate (yellow arrow); (**c**) chemical composition of type 4 showing Mo- and V-rich carbides.

In the Wrought-HT, the martensite matrix is similar to that of DED-HT. However, the size distribution of the small precipitates is different. Carbide size analysis reveals two types of precipitates, namely type 3 with a size of 0.40~1.20 μm (Figure 4a) and type 4 with a size distribution of 0.10~0.20 μm (Figure 4b). In other words, Wrought-HT exhibits smaller precipitates than DED-HT. Type 3 precipitates are Cr-rich carbides, and type 4 precipitates are present as Mo- and V-rich carbides (Figure 4a,c). These carbides are expected to be Cr_7_C_3_, Mo_2_C, and VC, which have been reported for conventional wrought materials [5,29]. The characteristics of each precipitate type are summarized in Table 2.

**Figure 5:**
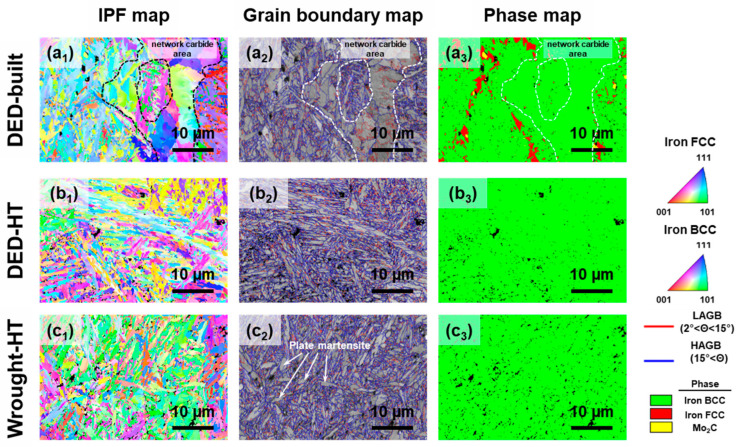
EBSD analysis results of DED-built, DED-HT, and Wrought-HT 5 wt% cold-work tool steels with (**a_1_**–**c_1_**) inverse pole figure maps, (**a_2_**–**c_2_**) grain boundary maps, and (**a_3_**–**c_3_**) phase maps.

Figure 5 shows the EBSD image analysis results for the DED-built, DED-HT, and Wrought-HT specimens. The inverse pole figure (IPF) and grain boundary map analysis results show that the DED-built material exhibits a microstructure comprising fine lath martensite and polygonal grains (Figure 5(a_1_,a_2_)). Phase map analysis confirms that the coarse grains around the carbide network are ferrite (Figure 5(a_3_)). Generally, polygonal ferrite is similar to equiaxed ferrite, with an aspect ratio of 1.5 or less [30]. In regions not adjacent to the network carbide, a large amount of retained austenite is identified (Figure 5(a_3_)). The volume fraction of retained austenite was measured to be 11.5 ± 3.0%. Additionally, a distribution of block-type Mo_2_C carbide with sizes ranging from 0.6 to 1.5 μm is observed along the austenite region. These analytical results suggest that the high cooling rate during the additive manufacturing process causes supersaturation of carbon at the prior austenite grain boundaries, which affects austenite stabilization and carbide formation. In contrast, DED-HT exhibits lath martensite throughout, whereas Wrought-HT shows microstructural features of both lath martensite and plate martensite (Figure 5(b_1_–b_3_),(c_1_–c_3_)). For the DED-built sample, an overall grain size including retained austenite and polygonal ferrite was 7.37 ± 6.42 μm. The average grain size used for martensite microstructure comparison among the DED-built, DED-HT, and Wrought-HT samples refers to the martensite block size, which is considered the effective grain size in hierarchical martensitic microstructures [31,32]. Martensite blocks were identified by applying a misorientation threshold of 55–65° [33,34]. The average martensite sizes of the three specimens are 1.12 ± 0.90, 1.29 ± 0.86, and 1.19 ± 0.88 μm for DED-built, DED-HT, and Wrought-HT, respectively. As a result, among the three 5 wt% Cr cold-work tool steel materials, DED-built with polygonal ferrite and austenite phases has the largest grain size, whereas Wrought-HT exhibits a relatively fine martensite block size compared to DED-HT.

### 3.2. Mechanical Properties

The Vickers hardness and room-temperature tensile results of the DED-built, DED-HT, and Wrought-HT samples are presented in Table 3. In terms of Vickers hardness, the DED-built specimen (702.5 HV) has hardness value similar to that of Wrought-HT (698.2 HV) and shows a slight decrease after post-heat treatment (650.5 HV, DED-HT).

**Figure 6:**
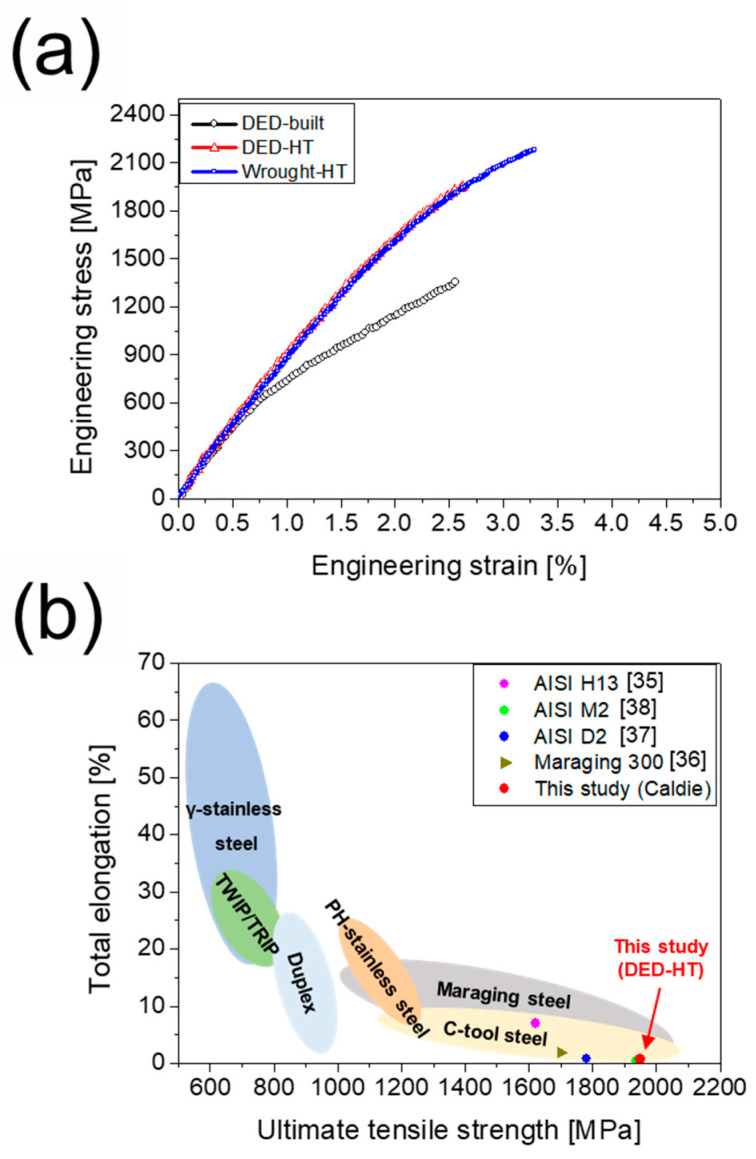
Room-temperature mechanical properties of DED-built, DED-HT, and Wrought-HT 5 wt% cold-work tool steels: (**a**) tensile stress–strain curves; (**b**) comparison of tensile properties between the DED-HT material and other high-strength steels fabricated by DED followed by post-heat treatment.

Figure 6a shows the room-temperature tensile stress–strain curves of the three materials. The yield strength (YS), ultimate tensile strength (UTS), and elongation (EL) of the DED-built specimens are 779.6 MPa, 1340.4 MPa, and 1.2%, respectively. The DED-HT, after post-heat treatment, shows a significant strength increase, with a YS of 1480.3 MPa, a UTS of 1949.4 MPa, and an EL of 0.7%. In contrast, the YS, UTS, and EL of Wrought-HT are 1520.7 MPa, 2229.6 MPa, and 1.0%, respectively, which are relatively higher than those of DED-HT. Figure 6b compares the tensile properties of the DED-HT material with those of other high-strength steels prepared using the DED method followed by post-heat treatment. The DED-HT sample demonstrates higher strength than DED-processed AISI H13 [35] and maraging 300 steel [36]. Furthermore, compared with high-carbon tool steels (AISI D2 and AISI M2) [37,38], it exhibits superior strength while maintaining similar elongation.

The DED-built sample exhibited the highest Kernel Average Misorientation (KAM) value (0.885), followed by the DED-HT (0.768) and Wrought-HT (0.724) samples. This trend suggests that the as-built microstructure retained a higher density of geometrically necessary dislocations (GNDs), due to the fresh martensite in the as-built sample may retain a high dislocation density, whereas in the DED-HT and Wrought-HT samples, post-heat treatment promoted the formation of tempered martensite, thereby reducing the dislocation density.

Although the DED-HT sample exhibited lower Vickers hardness compared to the DED-built sample, it showed a significantly higher tensile strength. The DED-built sample contains untempered fresh martensite, which retains high residual stresses and microstrain, contributing to elevated hardness. In contrast, the tempered martensite matrix in the DED-HT sample has undergone partial stress relief during heat treatment, reducing its hardness. Nevertheless, the overall mechanical performance, particularly tensile strength, is influenced not only by hardness but also by grain refinement, phase distribution, and matrix continuity. The presence of retained austenite, polygonal ferrite, and coarse grains in the DED-built sample tends to reduce its strength while increasing ductility, whereas the fine-grained martensitic matrix in the DED-HT condition enhances strength at the expense of elongation.

**Figure 7:**
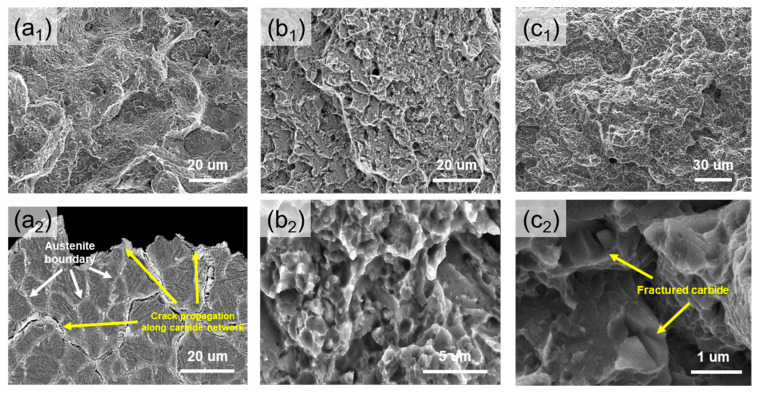
Tensile fractographies of the fractured samples in (**a**) DED-built, (**b**) DED-HT, and (**c**) Wrought-HT: (**a_1_**–**c_1_**) low magnification images, (**a_2_**) cross-sectional observation results, and (**b_2_**,**c_2_**) high magnification images.

The results of SEM analysis of the fracture surfaces of the DED-built, DED-HT, and Wrought-HT materials after tensile testing are shown in Figure 7. In the DED-built material, cracks propagate along the regions expected to be boundaries (prior austenite grain boundary and continuous carbide network boundary) and exhibit quasi-cleavage facets characteristic of brittle fractures (Figure 7(a_1_)). This is further confirmed by the cross-sectional image results (Figure 7(a_2_)), which show that numerous cracks occur along the continuous carbide network and prior austenite grain boundary regions. For both the DED-HT and Wrought-HT materials, crack propagation is observed along the prior austenite grain boundary (Figure 7(b_1_,c_1_)), whereas higher magnification observations reveal that the fracture mainly occurs along the martensite block and carbide (Figure 7(b_2_,c_2_)). DED-HT exhibits several cleavage fracture features that are characteristic of brittle fractures, which are discussed in more detail later. However, in Wrought-HT, fractured carbides are observed on the fracture surface. Generally, when tool steel is loaded, carbides preferentially absorb load energy and delay fracture [39,40]. From the above fracture results, Wrought-HT shows a more dominant ductile fracture behavior than DED-HT, which corresponds well with the higher elongation observed in the tensile results.

## 4. Discussion

### 4.1. Solidification Model of DED-Built Material

**Figure 8:**
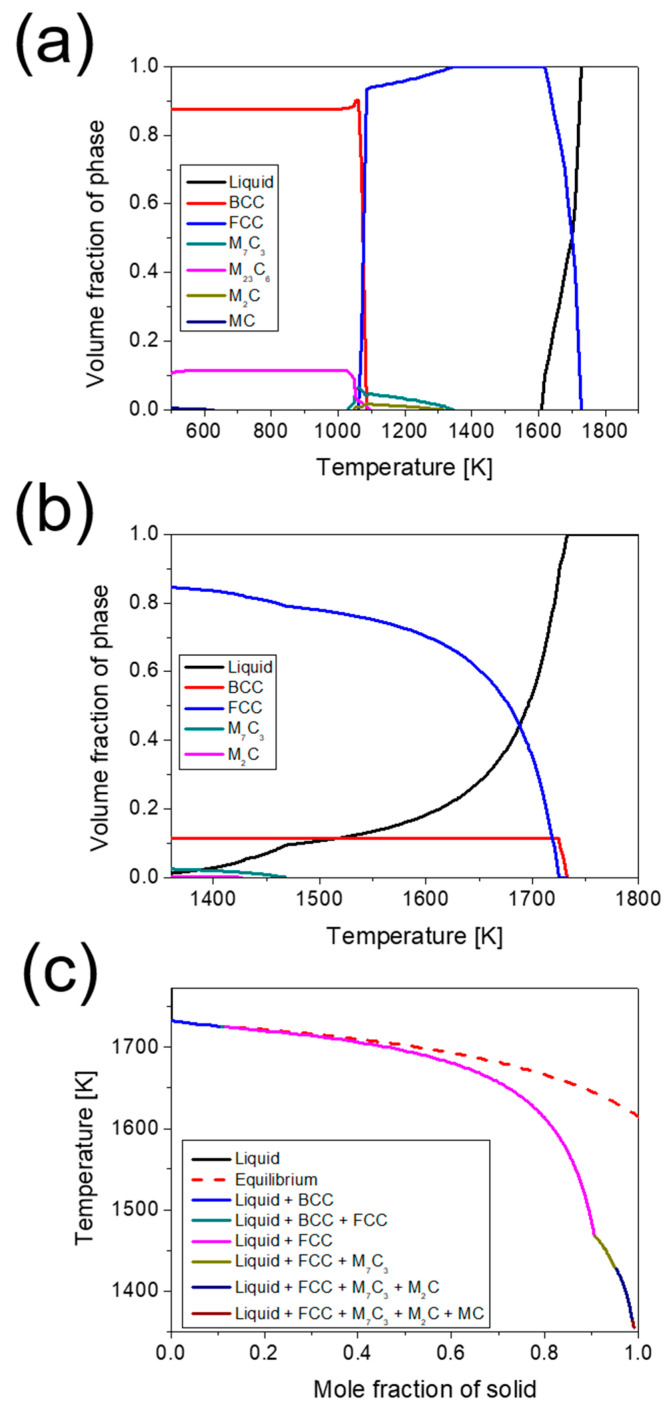
Calculated phase volume fractions of 5 wt% cold-work tool steel: (**a**) equilibrium state, (**b**) Scheil–Gulliver solidification model used in this study, and (**c**) mole fraction of the solid from the liquid state during solidification.

The equilibrium and non-equilibrium phase diagrams and solidification models calculated using the Thermo-Calc software are presented in Figure 8. In the equilibrium solidification model (Figure 8a), as the temperature decreases in the liquid-phase region, M_7_C_3_ and M_2_C carbides precipitate from the FCC matrix, and M_23_C_6_ carbide begins to form with a phase transformation to BCC at 1080~1100 K. The non-equilibrium solidification shown in Figure 8b,c was predicted using the Scheil–Gulliver model. This suggests that the primary solid phase, BCC, is formed first during solidification, followed by FCC. Based on the above results, the preferentially formed BCC and FCC phases at high temperatures are expected to be delta ferrite and austenite, respectively. However, the Scheil–Gulliver model predicts diffusional phase transformations, and phase transformations depending on the thermal history formed in the AM process should be considered additionally [41]. The delta ferrite predicted to form in the primary solidification state, as shown in the Scheil–Gulliver model, is not identified in the actual as-built microstructure. The ferrite in the microstructure is expected to form in a subsequent phase rather than in the primary solidification state. This is attributed to the preferential formation of thermodynamically stable austenite at high solidification rates [42,43,44]. In solidification modes with high cooling rates, austenite exhibits a higher dendrite tip temperature than delta ferrite. The difference between the dendrite tip temperature and liquidus temperature is lower for the austenite phase, indicating a relatively higher possibility of austenite formation [42,45]. After austenite formation, the alloying elements in the residual-melt region become localized through rapid solidification, inducing segregation and carbide formation. In particular, an increase in the FCC and M_7_C_3_ carbides is observed at 1468 K, with a simultaneous decrease in the liquid-phase region. This can be understood as the formation of the FCC phase (retained austenite) and M_7_C_3_ owing to the eutectic reaction in the liquid-phase region during solidification. Subsequently, M_7_C_3_, M_2_C, and MC carbides are formed sequentially.

### 4.2. Microstructural Evolution During DED Processing and Post-Heat Treatment

**Figure 9:**
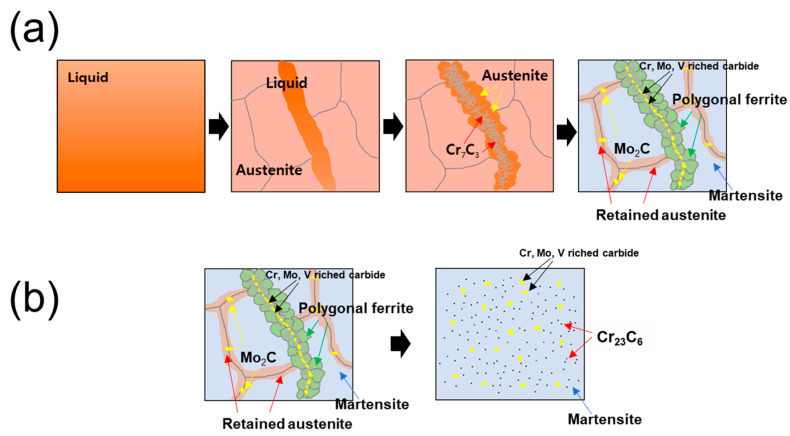
Schematic of the microstructure evolution in DED 5 wt% cold-work tool steel: (**a**) solidification in deposition process and (**b**) after post-heat treatment.

Based on microstructure observations and the Scheil–Gulliver solidification model results, a schematic of the expected microstructure evolution during the building process and post-heat treatment is shown in Figure 9. As the cooling progresses, the residual liquid-phase region begins to solidify, preferentially forming prior austenite owing to the supersaturated carbon. Owing to the high solidification rate, regions with a strong segregation of alloying elements may have a lower melting point than their surroundings and still exist as a localized liquid phase during solidification [46,47]. These liquid-phase regions form a eutectic structure after solidification through a eutectic reaction. Because of the high cooling rate in the eutectic reaction, sufficient time is not provided for the growth of the carbide. Consequently, the carbide is formed at a microscopic size where growth is inhibited [48]. In addition, the residual liquid phase, which does not participate in the growth of carbides during the eutectic reaction, is transformed into austenite. The eutectic reaction consists of austenite and Cr_7_C_3_ (lamellar-type carbide), followed by the formation of block-type carbides, such as Cr_7_C_3_, Mo_2_C, and VC. Multiple carbide precipitates reduce the carbon content in the austenite formed by the eutectic reaction and increase its instability [49]. Additionally, heat transfer from the newly melted layer to the previous layer causes a temperature increase, representing the effect of repeated in situ tempering during the process [50,51]. This complex thermal history and instability of austenite can consequently lead to the possibility of an austenite-to-ferrite transformation owing to the low cooling rates at high temperatures [52,53]. Meanwhile, in the region of the prior austenite boundary, differences in the phase stability occur owing to element segregation. During solidification, segregation due to carbon concentration and a relatively low martensite starting temperature (M_S_) are induced at the austenite grain boundary, which locally increases the thermodynamic stability of austenite and can lead to the formation of additional carbides [42]. However, the unstable austenite internal regions reach the M_S_ during rapid solidification and are transformed into martensite. After the post-heat treatment, the material shows a microstructural characterization in which the carbides are uniformly distributed in the tempered martensite matrix. Herein, the carbides exhibit the following two distinctive features: (1) Cr_23_C_6_ formed with increased C and Cr concentrations in the matrix owing to the dissolution of Cr_7_C_3_, and (2) Cr-, Mo-, and V-rich carbides change from a network to a separated form.

### 4.3. Effect of Hierarchical Structure and Precipitate on Tensile Property

**Figure 10:**
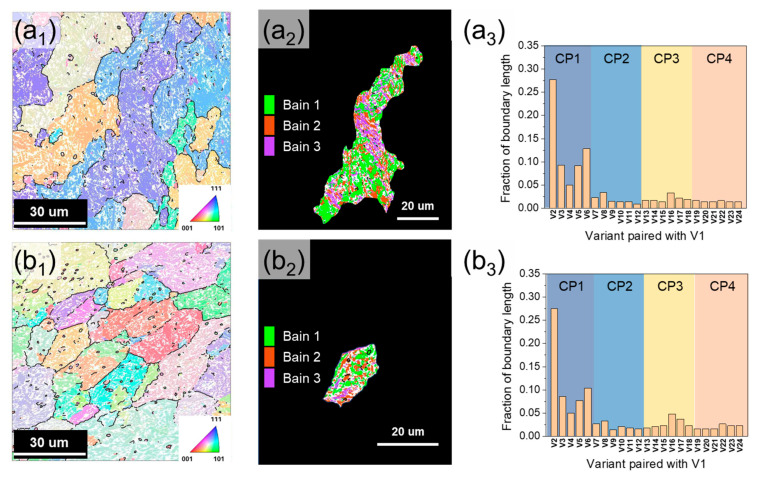
Reconstructed prior austenite grain results of (**a**) DED-HT and (**b**) Wrought-HT: (**a_1_**,**b_1_**) inverse pore figure maps indicating PAG size difference between DED-HT and Wrought-HT, (**a_2_**,**b_2_**) Bain group distribution mapping in single PAG area, and (**a_3_**,**b_3_**) 24 variant fraction according to the K–S relationship.

The phase transformation from prior austenite grain (PAG) to lath martensite can be described based on the Kurdjumov–Sachs (K–S) relationship ({111}γ//{110}α, <110>γ//<111>α), which is categorized into 24 different orientation relationships [54]. These 24 variants can be grouped into close-packed parallel planes (CP groups) with the same habit plane or Bain groups with similar crystallographic orientations. Martensite packets and blocks are composed of variants within the same CP and Bain groups, respectively [55,56]. The relationship between the prior austenite grain (PAG) size and the variant is an important microstructural factor in interpreting the mechanical properties and crack propagation. Prior austenite reconstruction was performed by applying Mtex (v5.1.1) and MATLAB (R2019a) codes based on the 24 K-S OR variants summarized in Table 4. Figure 10a_1_,b_1_ shows the reconstructed PAGs of DED-HT and Wrought-HT, respectively. The average size of the formed PAGs is 61 ± 35 μm in DED-HT and 35 ± 28 μm in Wrought-HT. Wrought-HT shows a smaller PAG size. Generally, a smaller PAG size leads to a reduction in the size of packets, blocks, and laths, which can significantly contribute to strength improvements [57,58].

Figure 10(a_2_,b_2_) reveals the distribution of the Bain groups in each single PAG grain of DED-HT and Wrought-HT, and compared to DED-HT, Wrought-HT showed a more uniform distribution of Bain groups. This feature is related to the PAG size. In general, when the PAG size is reduced, the limited dislocation slip cannot sufficiently accommodate the transformation-induced stress, resulting in the formation of multiple martensite variants that reduce the internal strain (self-accommodation) [59,60]. This, in turn, provides the driving force for a uniform Bain group distribution. The average size of the carbide precipitated by the post-heat treatment is relatively larger for DED-HT (type 1: 1.05 μm, type 2: 0.22 μm) compared with Wrought-HT (type 3: 0.81 μm, type 4: 0.15 μm). The high-angle block boundary (>45°) of the CP 1 group exhibits a higher fraction in DED-HT than in Wrought-HT (Figure 10a_3_,b_3_). The size of the precipitates is influenced by the high carbon diffusion rate at the high-angle block boundaries during tempering [61]. Consequently, this may be responsible for the formation of relatively large precipitates during DED-HT. In contrast, the average martensite block sizes are 1.26 and 1.19 μm in DED-HT and Wrought-HT, respectively, indicating that Wrought-HT exhibits a relatively finer block size.

**Figure 11:**
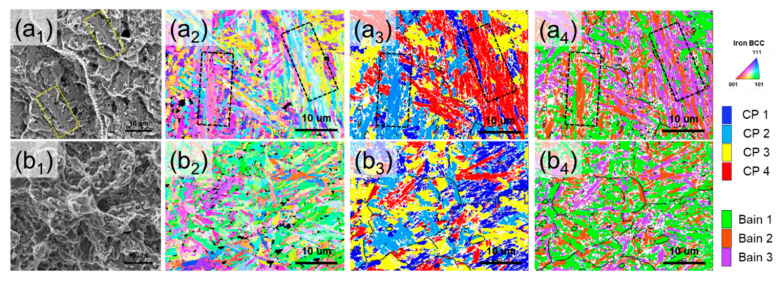
Relationship between tensile fractography and hierarchical sub-grain in (**a**) DED-HT and (**b**) Wrought-HT: (**a_1_**, **b_1_**) fractography after tensile test, (**a_2_**, **b_2_**) inverse pore figure maps in initial microstructure, (**a_3_**, **b_3_**) CP group maps, and (**a_4_**, **b_4_**) Bain group maps. Dotted box area shows the cleavage fracture caused by the low-angle grain boundary formed between variant regions of the same Bain group.

**Figure 12:**
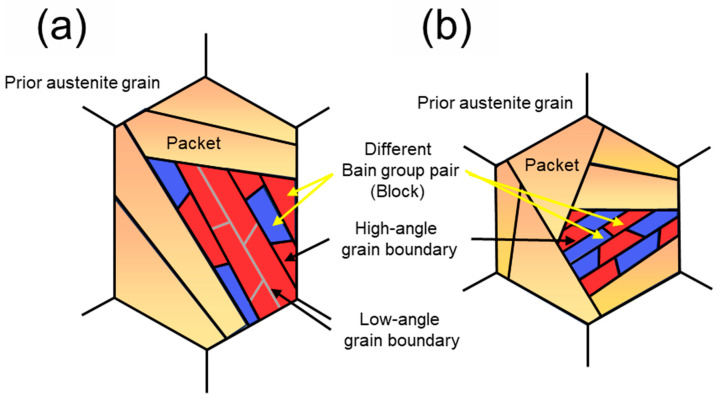
Schematics of variant structures of martensites: (**a**) DED-HT and (**b**) Wrought-HT.

To compare the fracture features of DED-HT and Wrought-HT and their relationship with the hierarchical microstructure, the tensile fractography SEM images and IPF maps, CP group and Bain group maps of the initial microstructures are shown in Figure 11. In DED-HT, dense CP groups (packet grains) grow along the build direction. Within these CP groups, multiple variant regions (block grains) belonging to the same Bain group are characterized by adjacent features (regions enclosed by the black dotted box in Figure 11(a_2_–a_4_)). High-angle grain boundaries form at different Bain group variant boundaries, whereas low-angle grain boundaries form between the variant regions of the same Bain group [62]. Compared to high-angle grain boundaries, low-angle grain boundaries are more prone to crack propagation, leading to their expansion [63]. These regions of low-angle grain boundary concentration in the DED-HT indicate the potential for brittle fracture through cleavage fracture formation (region enclosed by the yellow dotted box), as shown in Figure 11a_1_. The formation and propagation of these cleavage cracks can be accelerated by the presence of coarser carbides than those in Wrought-HT. In contrast, Wrought-HT is mostly composed of boundaries between variant regions belonging to different Bain groups, which are expected to inhibit crack propagation to some extent (Figure 11(b_2_–b_4_)). Figure 12 shows the above results and presents a schematic of the microstructure distribution inside the PAG of DED-HT and Wrought-HT.

### 4.4. Strengthening Mechanism

For carbon tool steels with tempered martensite, the strengthening factors affecting the yield strength are primarily solid-solution strengthening, precipitation strengthening, grain refinement, and dislocation strengthening. These mechanisms can be represented by the following equation [64].

(1) $$\sigma_{y}=\sigma_{0}+\sigma_{ss}+\sigma_{g}+\sigma_{d\;}+\sigma_{p}\;$$

where $\sigma_{0}$ is the lattice friction stress with a value of 54 MPa for Fe atoms [65]. $\sigma_{ss}$ represents solid solution strengthening, $\sigma_{g}$ represents grain refinement strengthening, $\sigma_{d\;}$ represents dislocation strengthening, and $\sigma_{p}$ represents precipitation strengthening.

Solid-solution strengthening can be divided into the interstitial strengthening of C atoms in the Fe matrix and substitutional strengthening from other alloying elements, such as Cr, Mo, Mn, Si, and V. The equations for each type of solid-solution strengthening are as follows [64,66]:

(2) $$\sigma_{ss,\;i}=1171.3X_{C}^{1/3}$$

(3) $$\sigma_{ss,s}=K_{\;i}X_{\;i}$$

where $X_{C}$ is the concentration (at.%) of the C atom, $K_{\;i}$ is the strengthening coefficient per 1 at.% of alloying element, and $X_{\;i}$ is the concentration (at.%) of substitutional atoms. The concentration of each element was measured using TEM-EDS analysis of the martensite lath region. The results showed a value of 351 MPa for $\sigma_{ss,\;i}$ in DED-HT and 376 MPa in Wrought-HT. While TEM-EDS is practical for localized compositional analysis, it is known to have limited sensitivity and accuracy for quantifying light elements, such as carbon in metallic matrices. Techniques, like atom probe tomography (APT), could provide more precise solute carbon measurements but were not employed in this study. Thus, the calculated $\sigma_{ss,\;i}$ should be considered as an approximate value, with recognition of the potential uncertainties introduced by the measurement method. The $K_{\;i}$ values for Mo, Cr, V, Si, and Mn elements used to calculate $\sigma_{ss,s}$ were 15.90, 2.60, 2.00, 25.80, and 16.90, respectively, resulting in calculated $\sigma_{ss,s}$ values of 51 MPa for DED-HT and 62 MPa for Wrought-HT. The predominance of plate martensite observed in the Wrought-HT sample is attributed to its higher carbon content (0.89 wt.%) compared to the DED-HT sample (0.72 wt.%), as confirmed by TEM-EDS analysis. Wide plate-shaped martensite is advantageous in accommodating lattice strain due to increased carbon content [67,68]. This result is consistent with the higher volume fraction of carbides observed in the DED-HT sample, suggesting that more carbon was consumed during carbide formation.

The effect of grain refinement on the increase in the yield strength was calculated using the Hall–Petch formula [69], as follows:

(4) $$\sigma_{g}=k_{y}/\sqrt{d}$$

A Hall–Petch coefficient ($k_{y}$) of 120 MPa·μm^1/2^ was employed to estimate the contribution of grain refinement strengthening [70,71,72]. This value is often used as a general approximation for martensitic steels containing alloying elements, although it is acknowledged that $k_{y}$ may vary depending on steel composition, grain morphology, and processing history. Thus, the present estimation should be considered an approximate reference. d is the effective grain size, and the width of the martensite lath is considered as the correct grain size parameter for strength gain. To obtain accurate results, this approach considers the different martensite lath widths found in complex microstructures composed of laths and plate martensite. The measured lath widths were 165 and 110 nm for DED-HT and Wrought-HT, respectively, and the values of $\sigma_{g}$ derived by substituting into Equation (4) were 295 and 361 MPa for DED-HT and Wrought-HT, respectively.

The increase in strength owing to dislocation strengthening can be expressed as follows [73]:

(5) $$\sigma_{d}=M\alpha Gb\sqrt{\rho }$$

where M is the Taylor factor (3), α is a constant (0.25), G is the shear modulus (76 GPa), and b is the Burgers vector (0.248 nm). ρ is the dislocation density, which was calculated by the following Equation (6) [73,74,75]:

(6) $$\rho =\frac{\alpha \theta }{bd}$$

where α is a constant set to 3, depending on the geometrical structure of the dislocation array [76]. θ is the average misorientation angle, b is the Burgers vector, and d is the average distance of dislocation boundaries (step size of the EBSD map) [75]. The θ, b*,* d values for Equation (6) are 0.01341 radian, 0.248 nm, 90 nm for DED-HT and 0.01265 radian, 0.248 nm, 90 nm for Wrought DED-HT, respectively. Based on the experimental results, we obtained dislocation densities of 1.80 × 10^15^ m^−2^ and 1.39 × 10^15^ m^−2^ for DED-HT and Wrought-HT, respectively. Consequently, the dislocation strengthening was predicted to be 600 (DED-HT) and 582 MPa (Wrought-HT).

The strength effect due to precipitation strengthening can be expressed using the Ashby–Orowan equation [77], as follows:

(7) $$\sigma_{p}=0.1864M\frac{Gb}{v}\frac{V_{f}^{1/2}}{d}ln(\frac{1.2d}{2b})$$

where v is the Poisson’s ratio (0.291 [47]) and $V_{f}$ is the volume fraction of the precipitates. $V_{f}$ is calculated as follows [78]:

(8) $$V_{f}=\frac{1.4\pi }{6}\frac{N_{i}d_{i}^{2}}{A}$$

where $N_{i}$ is the number of precipitates and $d_{i}$ is the average diameter of the precipitates.

For $N_{i}$, the number of precipitates existing in an area of 100 μm^2^ (10 μm × 10 μm) was considered, and the values of 3.3 and 56.2 for type 1 and 2 precipitates of DED-HT, respectively, and 4.5 and 78.8 for type 3 and 4 precipitates of Wrought DED-HT, respectively, were applied. Herein, the precipitate fraction of DED-HT was 0.045 (type 1: 0.024, type 2: 0.021), which was higher than that of Wrought-HT with 0.033 (type 3: 0.020, type 4: 0.013). As a result, precipitation strengthening was calculated to be 194 (DED-HT) and 218 MPa (Wrought-HT). However, the results suggested that the relatively smaller precipitate size of Wrought-HT had a greater influence on the increase in strengthening than the precipitate fraction.

**Figure 13:**
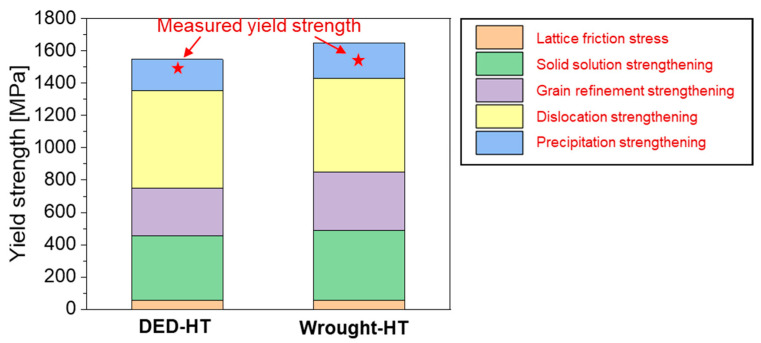
Strengthening contributions in yield strengths calculated by applying various strengthening mechanisms compared with the experimental results.

Figure 13 compares the sum of the predicted yield strength values calculated by reflecting each strengthening mechanism with the experimentally measured values. The predicted values are similar to the actual measured values, with the strengthening factors contributing to the strength in the following order: dislocation, solid solution, grain refinement, and precipitation, which are common to both DED-HT and Wrought-HT. Comparing the two materials, dislocation density strengthening dominates in DED-HT, owing to the formation of a barrier to dislocation slip through the formation of a high-angle block boundary. In contrast, in Wrought-HT, the strengthening factor is dominated by fine grains, interstitial carbon strengthening in the martensite laths, and small carbides.

While the present study confirms the technical feasibility of DED for high-carbon tool steels, its industrial deployment requires further evaluation of economic and scalability factors. The ability of DED to enable localized deposition and repair offers distinct advantages in tool steel applications where components experience localized damage or wear. This can reduce material waste and maintenance costs, providing cost-efficient alternatives to conventional fabrication methods. As a result, Future studies should focus on evaluating large-scale manufacturability, optimizing post-processing strategies, and assessing the cost–performance of DED in production-scale scenarios.

## 5. Conclusions

In this study, a crack-free 5 wt% Cr cold-tool steel was fabricated via DED, and its microstructure and mechanical properties were investigated after heat treatment; the following conclusions were obtained:

1. DED-built comprised fine lath martensite, retained austenite, and polygonal ferrite, and a carbide network characterized by the distribution of Cr-rich lamellar-type carbides around the Cr-, V-, and Mo-rich block-type carbides was identified. DED-HT and Wrought-HT exhibited a martensitic matrix, and small precipitates were uniformly distributed within the matrix. The average grain sizes of DED-built, DED-HT, and Wrought-HT were 7.37 ± 6.42, 1.29 ± 0.86, and 1.19 ± 0.88 μm, respectively.
2. DED-built (YS: 779.6 MPa, UTS: 1340.4 MPa, EL: 1.2%) showed a significant strength increase after heat treatment (DED-HT-YS: 1480.3 MPa, UTS: 1949.4 MPa, EL: 0.7%). In contrast, in Wrought-HT, YS, UTS, and EL were obtained as 1520.7 MPa, 2229.6 MPa, and 1.0%, respectively, exhibiting slightly better properties than those of DED-HT.
3. The non-equilibrium solidification of 5 wt% Cr cold-work tool steel was predicted using the Scheil–Gulliver model. After austenite formation in the liquid-phase region, a eutectic reaction (austenite + Cr_7_C_3_) was proposed in the residual liquid-phase region. The subsequent formation of successive carbides increased the instability of austenite, and it was predicted that the eutectic austenite was transformed into ferrite by the repeated heat-treatment effect of in situ tempering during the process. It was proposed that the interior region, which is more unstable than the prior grain boundary in the prior austenite region, transformed into martensite during solidification.
4. Wrought-HT exhibited relatively finer PAG, blocks, and laths, along with a more uniform distribution of Bain groups compared with DED-HT. In DED-HT, the low-angle grain boundaries between variants of the same Bain group facilitated crack propagation and brittle fracture. By contrast, Wrought-HT predominantly consisted of variants from different Bain groups, which inhibited crack propagation.
5. In DED-HT, the strengthening factor due to high dislocation density was more dominant than that in Wrought-HT. Compared with DED-HT, the strengthening factor due to small grains, carbon interstitial strengthening in the martensite lath, and small carbides was higher in Wrought-HT. In the case of precipitation strengthening, the carbide size had a greater effect on the strengthening effect than the carbide fraction.

## Figures and Tables

**Table 1:** Chemical compositions of as-built and wrought 5 wt% Cr cold-work tool steels.

Element (wt%)	Fe	C	Si	Mn	Cr	Mo	V
As-built	Bal.	0.65	0.21	0.54	4.64	2.29	0.42
Wrought	Bal.	0.64	0.19	0.48	4.72	2.26	0.42

**Table 2:** Characteristics of precipitate types in DED-HT and Wrought-HT materials.

Material	Precipitate Type	Chemical Composition/(Phase)	Size Distribution (μm)
DED-HT	Type 1	C, Cr, Mo, V	0.70~1.20
	Type 2	C, Cr (Cr_23_C_6_)	0.15~0.25
Wrought-HT	Type 3	C, Cr (Cr_7_C_3_)	0.40~1.20
	Type 4	C, Mo, V (Mo_2_C_,_ VC)	0.10~0.20

**Table 3:** Mechanical properties of DED and wrought 5 wt% cold-work tool steel materials according to post-heat treatments.

	Vickers Hardness [HV]	Yield Strength [MPa]	Ultimate Tensile Strength [MPa]	Elongation [%]
DED-built	702.5 ± 13.7	779.6 ± 14.4	1340.4 ± 17.0	1.2 ± 0.2
DED-HT	650.5 ± 12.0	1480.3 ± 28.2	1949.4 ± 31.6	0.7 ± 0.1
Wrought-HT	698.2 ± 10.2	1520.7 ± 19.1	2229.6 ± 24.2	1.0 ± 0.1

**Table 4:** Twenty-four variants in the K–S relationship (reproduced from [55] with permission Elsevier).

Variant No.	Plane Parallel	Direction Parallel	Bain Group	CP Group	Misorientation from V1 (°)
V1	(111)_γ_//(011)_α_	[$\bar{1}01$]_γ_//[$\bar{11}1$]_α_	B1	CP1	-
V2		[$\bar{1}01$]_γ_//[$\bar{1}1\bar{1}$]_α_	B2		60.0
V3		[$01\bar{1}$]_γ_//[$\bar{11}1$]_α_	B3		60.0
V4		[$01\bar{1}$]_γ_//[$\bar{1}1\bar{1}$]_α_	B1		10.5
V5		[1$\bar{1}0$]_γ_//[$\bar{11}1$]_α_	B2		60.0
V6		[1$\bar{1}0$]_γ_//[$\bar{1}1\bar{1}$]_α_	B3		49.5
V7	($1\bar{1}1$)_γ_//(011)_α_	[$10\bar{1}$]_γ_//[$\bar{11}1$]_α_	B2	CP2	49.5
V8		[$10\bar{1}$]_γ_//[$\bar{1}1\bar{1}$]_α_	B1		10.5
V9		[$\bar{11}0$]_γ_//[$\bar{11}1$]_α_	B3		50.5
V10		[$\bar{11}0$]_γ_//[$\bar{1}1\bar{1}$]_α_	B2		50.5
V11		[011]_γ_//[$\bar{11}1$]_α_	B1		14.9
V12		[011]_γ_//[$\bar{1}1\bar{1}$]_α_	B3		57.2
V13	($\bar{1}11$)_γ_//(011)_α_	[$0\bar{1}$1]_γ_//[$\bar{11}1$]_α_	B1	CP3	14.9
V14		[$0\bar{1}$1]_γ_//[$\bar{1}1\bar{1}$]_α_	B3		50.5
V15		[$\bar{1}0\bar{1}$]_γ_//[$\bar{11}1$]_α_	B2		57.2
V16		[$\bar{1}0\bar{1}$]_γ_//[$\bar{1}1\bar{1}$]_α_	B1		20.6
V17		[110]_γ_//[$\bar{11}1$]_α_	B3		51.7
V18		[110]_γ_//[$\bar{1}1\bar{1}$]_α_	B2		47.1
V19	($11\bar{1}$)_γ_//(011)_α_	[$\bar{1}10$]_γ_//[$\bar{11}1$]_α_	B3	CP4	50.5
V20		[$\bar{1}10$]_γ_//[$\bar{1}1\bar{1}$]_α_	B2		57.2
V21		[$0\bar{11}$]_γ_//[$\bar{11}1$]_α_	B1		20.6
V22		[$0\bar{11}$]_γ_//[$\bar{1}1\bar{1}$]_α_	B3		47.1
V23		[101]_γ_//[$\bar{11}1$]_α_	B2		57.2
V24		[101]_γ_//[$\bar{1}1\bar{1}$]_α_	B1		21.1

## Data Availability

The original contributions presented in this study are included in the article. Further inquiries can be directed to the corresponding author.
